# Efficacy of Paclitaxel plus TS1 against previously treated *EGFR* mutated non-small cell lung cancer

**DOI:** 10.7717/peerj.7767

**Published:** 2019-09-24

**Authors:** Yen-Han Tseng, Jen-Fu Shih, Heng-Sheng Chao, Yuh-Min Chen

**Affiliations:** 1 School of Medicine, National Yang-Ming University, Taipei, Taiwan; 2 Department of Chest Medicine, Taipei Veterans General Hospital, Taipei, Taiwan; 3 Division of Pulmonary Medicine, Department of Internal Medicine, Shuang Ho Hospital, Taipei Medical University, New Taipei City, Taiwan; 4 Center of Excellence for Cancer Research, Taipei Medical University, Taipei, Taiwan

**Keywords:** Non-small cell lung cancer, Epidermal growth factor receptor (*EGFR*), TS1

## Abstract

**Background:**

Later line chemotherapy (≥2nd lines) such as Docetaxel or immunotherapy is frequently used. As the life expectancy of lung cancer patients is getting longer, we need to provide more treatment options. Other treatment options are not well documented except for Doxetaxel and immunotherapy. Therefore, the efficacy of paclitaxel plus TS1 (TTS1) is warranted.

**Methods:**

We retrospectively reviewed the chart records of our non-small cell lung cancer patients who were treated between 2010 and 2013. Clinical characteristics, type of tumor, EGFR mutation status, and treatment response to first-line EGFR-TKI therapy and efficacy of TTS1, were collected.

**Results:**

Twenty eight patients were enrolled in this study. No patients archived complete response and seven patients had partial response (ORR: 25%). The disease control rate was 60.7% (17/28). The progression free survival (PFS) was 4.0 months and overall survival (OS) was 15.8 months. Of them, 17 had EGFR mutations, eight *EGFR* wild type, and three were unknown EGFR status. After TTS1 treatment, patients with *EGFR* mutations had better PFS (4.9 months vs. 1.8 months) and OS (15.5 months vs. 7.2 months) compared with those of *EGFR* wild type.

**Conclusions:**

TTS1 are effective later line chemotherapy, especially in tumor *EGFR* mutated patients. Paclitaxel plus TS1 is another treatment of choice for NSCLC patients before a more effective treatment strategy is found.

## Introduction

Median survival time of patients with metastatic non-small cell lung cancer (NSCLC) has increased in recent two decades after usage of molecular targeted therapy and immunotherapy (Hamid et al., 2019; Planchard et al., 2019). However, systemic chemotherapy is still very important for majority of patients (Baxevanos & Mountzios, 2018). In addition, chemotherapy is frequently used as bridge between targeted therapy, or combination with targeted therapy or immunotherapy, and is still the mainstay for heavily treated patients. As for regimens of chemotherapy, previous studies showed combination therapy is better than single agent treatment for NSCLC patients (Lilenbaum et al., 2009; Morth & Valachis, 2014).

Paclitaxel is a well-known cytotoxic agent which would enhance polymerization of tubulin and stabilize microtubules. This mechanism therefore could induce mitotic arrest and cause apoptosis of tumor cells (Dziadyk et al., 2004; Horwitz, 1994). The regimens of paclitaxel based chemotherapy are widely used in NSCLC patients (Bunn Jr, 1996; Ramalingam & Belani, 2004). However, the regimen of paclitaxel combining with TS-1 is not well studied yet.

TS-1 is an oral anticancer agent that contained tegafur, gimeracil, and oteracil potassium (Ohba et al., 2009; Yoshioka et al., 2013). As a pro-drug of 5-FU, the mechanism is to inhibit DNA synthesis during S phase of the cell cycle (Focaccetti et al., 2015). TS-1 combined with cisplatin or carboplatin has been studied for NSCLC. The result showed that the regimen is similar to cisplatin plus doxetaxel or carboplatin plus paclitaxel (Takeda, 2013). However, platinum combined 3rd generation chemotherapeutic agents is the standard of care for non-small cell lung cancer, which mad platinum not available in later line chemotherapy regimen (Fossella et al., 2003; Gao et al., 2009; Genova et al., 2013). Based on the mechanism of each chemotherapeutic drug, TS1 is S phase specific and Paclitaxel is M phase specific. As the result, we combine these two drugs for patients with non-small cell lung cancer. In addition, most studies of TS1 were performed in Japan and there is only limited data available. The efficacy of TTS1 in Taiwan is not known. A bigger population size from other area is warranted, therefore.

The present retrospective study was designed to determine the efficacy of combination chemotherapy, paclitaxel plus TS1 (TTS1) in heavily treated NSCLC patients with or without *EGFR* mutation.

## Materials & Methods

### Study design and patients

We retrospectively reviewed and analyzed the chart records and image files of our lung cancer patients diagnosed between 1996 and 2017. Only those stage IV (American Joint Committee for Cancer staging system, 7th edition) NSCLC patients who had been treated previously and received paclitaxel plus TS1 were enrolled into the present study. The first-line treatment included tyrosine kinase inhibitor (TKI) for patients with *EGFR* mutation or *ALK* rearrangement and platinum-based chemotherapy for those who did not have *EGFR* or *ALK* mutations. Patients who had been treated with at least one line of platinum-based chemotherapy and with measurable disease; adequate bone marrow reserve with a WBC count ≥4,000/mm3, platelets ≥100,000/mm3 and hemoglobin ≥10 g/dL; and no previous history of paclitaxel nor TS-1 treatment. Patients with inadequate liver function (bilirubin >1.5 times above normal range, alanine transaminase (ALT) and aspartate transaminase (AST) > 3 times of normal), and inadequate renal function with creatinine >2.0 mg/dl were excluded from the treatment. The treatment consisted of paclitaxel 90 mg/m^2^ intravenous infusion on day 1 and daily TS-1 (80 mg for BSA<1.2 m^2^ , 100 mg for BSA 1.2–1.5 m^2^, and 120 mg for BSA>1.5 m^2^) from day 1 to day 7, every 2 weeks. With regard to dose modifications, the dose of paclitaxel was reduced to 80% and TS-1 reduced 20 mg if the absolute neutrophil count (ANC) was from 1.5 to 1.0 × 10^9^/L and/or the platelet count was from 99 to 75 × 10^9^/L on the day of the scheduled chemotherapy. The administration was delayed for one week if the ANC was below 1.0 × 10^9^/L or the platelet count below 75 × 10^9^/L. To evaluate the effectiveness of TTS1, we retrospectively reviewed the cohort from different perspectives. Clinical characteristics, including the patients’ age, gender, Eastern Cooperative Oncology Group (ECOG) performance status (PS), smoking history, type of driver mutations, were recorded. We compared the differences in basic patient characteristics and efficacy of TTS1 between patients with and without tumor *EGFR* mutations. This data review of the patients was approved by the institutional review board of Taipei Veterans General Hospital (VGHIRB No.: 2018-01-007AC).

### Efficacy evaluation

Chest computed tomography scan (including liver and adrenal glands) was performed within 3 weeks before starting chemotherapy, and every 2 to 3 months thereafter, or when confirmation of treatment response or disease progression was required. Types of response were assessed with the use of the Response Evaluation Criteria in Solid Tumors (RECIST version 1.1) (Eisenhauer et al., 2009). PFS was calculated from the date of administration of the first dose of TTS1 to the earliest sign of disease progression, as determined by means of the RECIST criteria, or death from any cause. Survival was measured from administration of the first dose of TTS1 until the date of death.

### *EGFR* mutation analysis

Two examination methods of *EGFR* mutation status were used. Patients were analyzed with Sanger DNA sequencing before the end of 2010. All the sequence variations were confirmed by multiple, independent polymerase chain reaction amplifications and repeated sequencing reactions. The majority of specimens were tested using the Scorpion amplification refractory mutation system method from 2011.

### Statistical analysis

All categorical variables were analyzed with ϰ^2^ tests. Mann–Whitney u test was conducted for continuous variables when comparing 2 groups. The chemotherapy response rate was compared between 2 groups. Median PFS and overall survival were calculated using the Kaplan–Meier method and compared by log-rank test. All statistical analyses were performed using SPSS software (version 19.0; SPSS Inc., Chicago, IL).

## Results

### Patients

**Figure 1:**
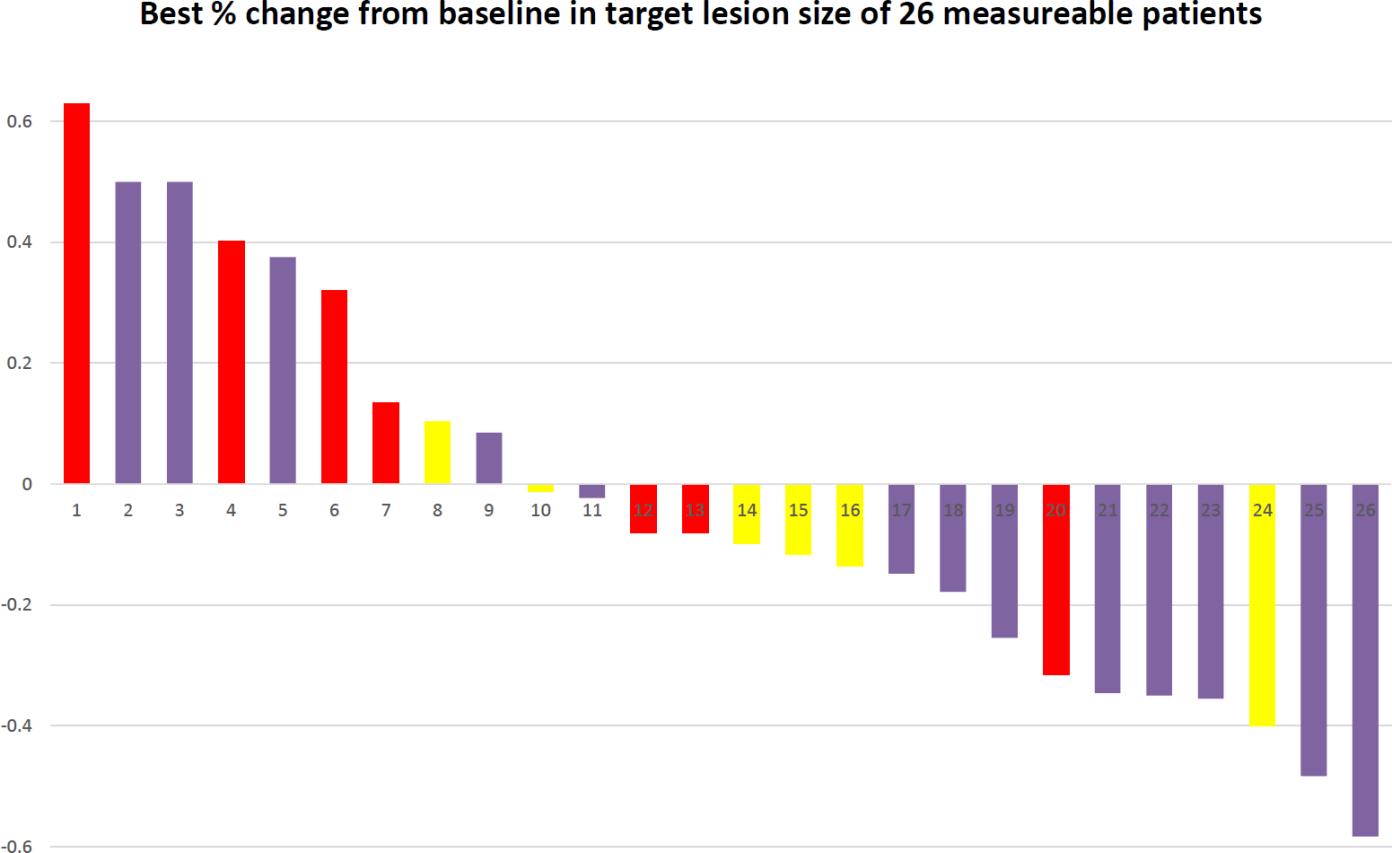
Waterfall plot. waterfall plot of best % change from baseline in target lesion size of 26.

There were 28 stage IV NSCLC patients enrolled in this study. One of them was squamous cell carcinoma and other 27 patients was adenocarcinoma. Age of the patients was between 39 years old to 84 years old. Patients’ clinical characteristics were shown in Table 1. After treatment, seven patients had partial response, 10 patients had stable disease, 10 patients had progressive disease, and the remaining 1 patient was unevaluable. The percentage of change from baseline in target lesion size of 26 measurable patients was shown in Fig. 1. Median PFS of these 28 patients was 4.0 months (95% CI [2.6–5.4] months), and median survival was 15.8 months (95% CI [10.9–20.6] months). Among all the patients, 17 patients had to get modulated dose due to low WBC count. The PFS is not significantly different between groups that adjusted dose and full dose (4.8 months vs. 4.2 months, *p* = 0.609). The OS between the two groups that adjusted dose and full dose were not different significantly, either (14.86 months vs. 15.18 months, *p* = 0.940). We sub-classify these patients according to previous treatment, which are chemotherapy alone and chemotherapy plus target therapy. Four patients received chemotherapy alone before TTS1 and 23 patients received chemotherapy and EGFR TKI before TTS1. The PFS and OS showed no significantly different. (PFS: 3.7 vs. 4.8 months; OS: 7.4 vs. 15.7 months).

**Table 1:** Driver mutations.

	All (*n* = 28)	EGFR mutation (*n* = 17)	Wild type (*n* = 8)	*p* value
Age	62.3 ± 11.5	60.3 ± 12.0	64.4 ± 10.4	0.382
Gender				0.389
Male	15 (53.6%)	11 (64.7%)	3 (37.5%)	
Female	13 (46.4%)	6 (35.3%)	5 (62.5%)	
Response				0.869
Partial response	7 (25.0%)	4 (23.5%)	2 (25%)	
Stable disease	10 (35.7%)	6 (35.3%)	2 (25%)	
Disease progression	10 (35.7%)	7 (41.2%)	4 (50%)	
Unevaluable	1 (3.6%)			
Disease control rate	17 (60.7%)	10 (58.8%)	4 (50%)	1.000
Treatment cycles	6.3 ± 3.0	6.1 ± 3.3	5.5 ± 3.1	0.630
Previous lines	5.4 ± 1.9	5.5 ± 1.9	4.6 ± 1.8	0.332
Line after TTS1	1.6 ± 1.2	1.9 ± 1.1	1.0 ± 1.1	0.057
PFS (months)	4.0 (2.6–5.4)	4.9 (3.2–6.6)	1.8 (0–3.9)	0.043
OS (months)	15.8 (10.9–20.6)	16.6 (10.6–22.6)	7.2 (0–14.3)	0.027

### Patients with *EGFR* mutation vs. patients without *EGFR* mutation

**Figure 2:**
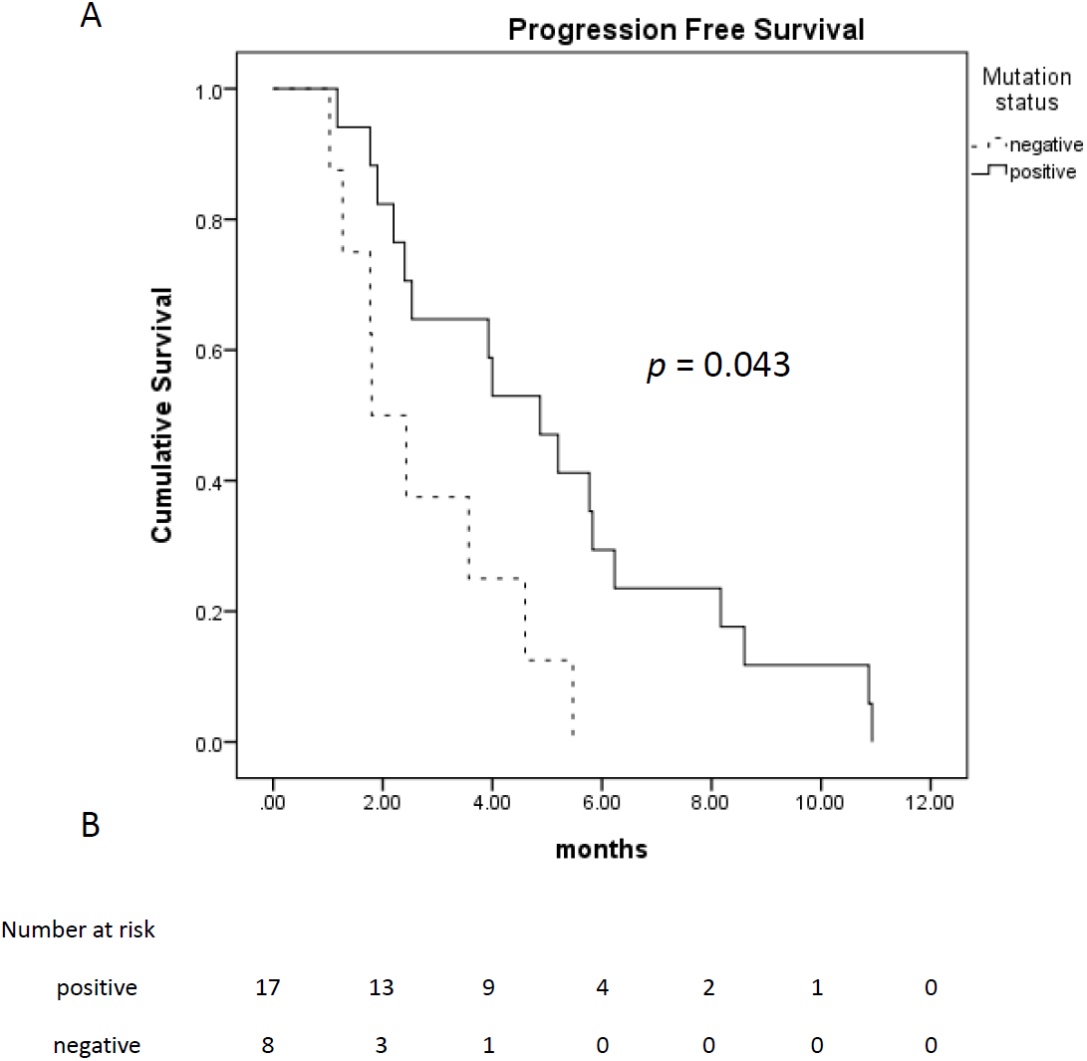
Progression free survival of patients who received TTS1. (A) Progression free survival of patients who received TTS1; (B) patient number that are alive.

**Figure 3:**
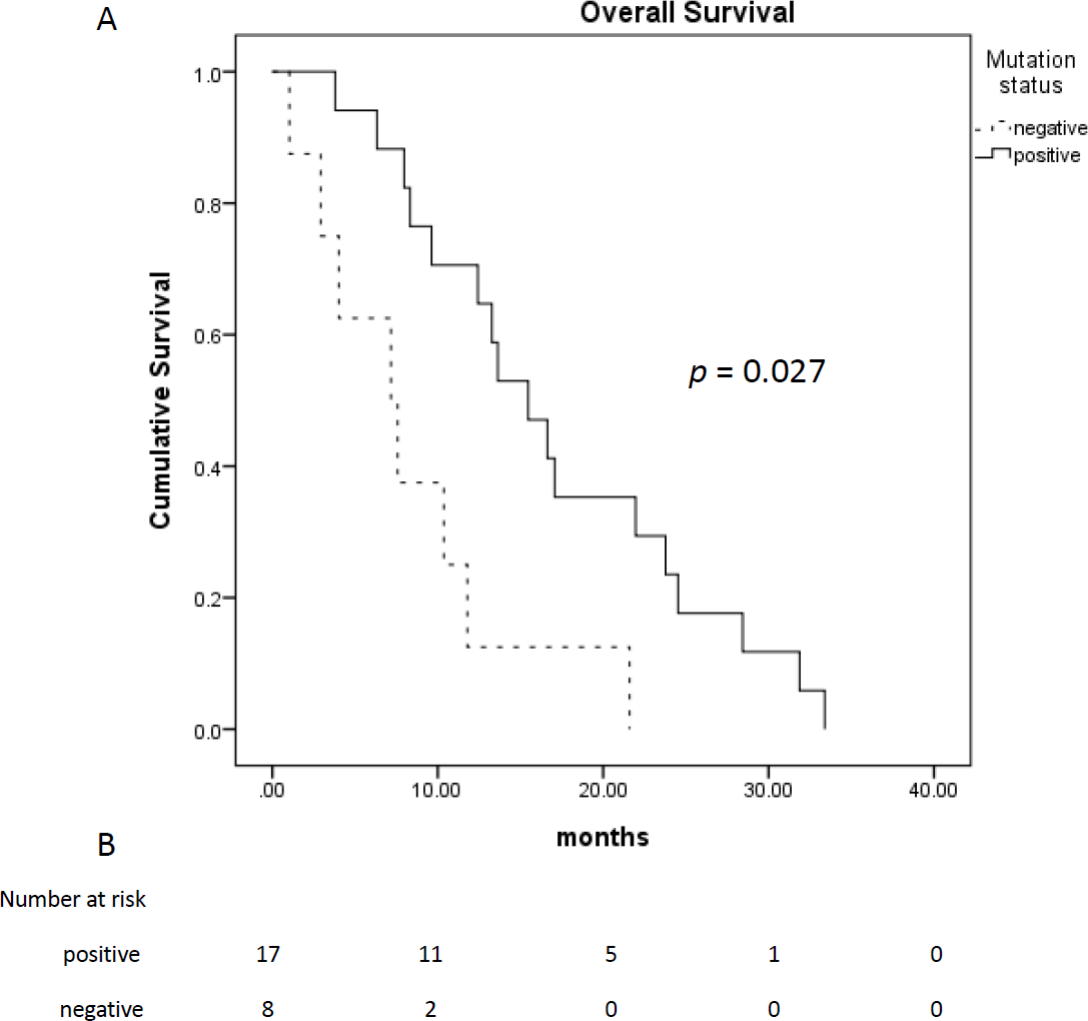
Overall survival of patients who received TTS1. (A) Overall survival of patients who received TTS1; (B) patient number that are alive.

Among 28 patients who received TTS1 treatment, 17 patients had *EGFR* mutations, eight patients were wild type, and remaining three patients were unknown *EGFR* status. The age and gender between those EGFR mutated or wild type groups were not statistically different. Treatment response and treatment cycles, were not statistically different, either. Patients of both groups received similar cycles of treatment before TTS1 (5.5 ± 1.9 cycles vs. 4.6 ± 1.8 cycles, *p* = 0.332) and after TTS1 (1.9 ± 1.1 cycles vs 1.0 ± 1.1, *p* =0.057). However, the PFS were longer in patients with *EGFR* mutations (4.9 months vs. 1.8 months, *p* = 0.043) (Fig. 2). The overall survival was better in EGFR mutation group, too (16.6 months vs. 7.2 months, *p* = 0.027) (Fig. 3) (Table 1).

### The responder of TTS1 has longer PFS although they have received more chemotherapy regimens previously

Twenty-eight patients received TTS1 therapy. Fifteen patients responded to TTS1, and 13 patients did not respond to TTS1. The general data including sex, gender, histology, performance status, and ratio of *EGFR* mutation status was not different (Table 2). Patients who responded to TTS1 usually received more cycles than those who did not respond to TTS1 (7.9 ± 2.9 cycles vs. 4.1 ± 1.8 cycles, *p* = 0.000). In addition, patients who responded to TTS1 received more previous treatments than those who did not respond to TTS1 (6.3 ± 1.7 lines vs. 4.4 ± 1.6 lines, *p* = 0.005). The OS of both groups was not significantly different (*p* = 0.15).

**Table 2:** Responsiveness of TTS1.

	Disease control (*n* = 15)	Disease progression (*n* = 13)	*p* value
Age	63.1 ± 10.8	61.4 ± 12.6	0.433
Gender			0.476
Male	7 (46.7%)	8 (61.5%)	
Female	8 (53.3%)	5 (38.5%)	
Histology			1.000
Adenocarcinoma	14 (93.3%)	13 (100%)	
Squamous cell carcinoma	1 (6.7%)	0 (0%)	
ECOG PS			0.139
0	1 (6.7%)	0 (0%)	
1	11 (73.3%)	6 (46.2%)	
2	3 (20.0%)	4 (30.8%)	
3	0 (0%)	3 (23.1%)	
Driver mutation	8 (66.7%)	9 (69.2%)	1.000
Treatment cycles	7.9 ± 2.9	4.1 ± 1.8	0.000
Previous lines	6.3 ± 1.7	4.4 ± 1.6	0.005
Line after TTS1	1.5 ± 1.2	1.7 ± 1.2	0.703
PFS (months)	4.8 (3.0–6.7)	1.9 (1.4–2.4)	0.002
OS (months)	12.4 (8.3–16.5)	13.3 (3.5–23.0)	0.150

## Discussion

Combination chemotherapy is more effective than single agent chemotherapy in lung cancer treatment (Bluthgen & Besse, 2015; Cheong et al., 2005; Lilenbaum et al., 2009). Paclitaxel is known to inhibit mitosis of tumor cells. The mechanism is to block the transition from G0 to S phase (Long & Fairchild, 1994). Initial study of single-agent paclitaxel for NSCLC patients was done with doses of 200–250 mg/m^2^. The response rate was 21–24% with PFS of 6-9 months. Subsequent studies administered lower dose of 175–225 mg/m^2^ and 135–200 mg/m^2^. The response rate ranged from 20–60% (Akerley 3rd, 2000; Socinski, 1999). TS-1, as a prodrug of 5FU, acts on S phase to inhibit DNA synthesis (Focaccetti et al., 2015). Until now, there were only limited data of TS-1 monotherapy for NSCLC patients. The synergic effect of TTS1 had been reported in gastric cancer treatment previously (Gotoh, Kawabe & Takiuchi, 2006; Mochiki et al., 2006; Sakurai et al., 2008). However, only few studies focus on the effect of TTS1 for NSCLC patients (Aono et al., 2012).

In our study, TTS1 demonstrated a high response rate and good PFS for heavily treated patients. Aono et al. started the regimen of paclitaxel ranged from 70-120 mg/m^2^ and TS1 of 80 mg/m^2^. Paclitaxel was used from intravenous infusion on day 1 and day 15, and TS1 was taken orally from day 1 to day 14. Then the patients take a rest for 2 weeks, which meant that the cycle was repeated every 4 weeks. The result showed that the response rate of TTS1 was 32.6% and disease control rate was 65.0% in previously treated NSCLC. 26.1% of their patients who received TTS1 as >4th line chemotherapy (Aono et al., 2012). In our study, paclitaxel was used on day 1 with the dose of 90 mg/m^2^ intravenous infusion, and TS-1 was took orally daily from day 1 to day 7 with the dose ranged from 80 to 120 according to the body weight of patients. Our data showed that the response rate was 25% and disease control rate was 60.7%. Compared with the result of *Aono* et al., our patients received much more chemotherapy regimens before the use of TTS1. A total of 25% of our patients received for more than six regimens before receiving TTS1. Therefore, our response rate and disease control rate is a little lower. The response rate of 2nd line chemotherapy is around 20–30% after 1st line EGFR TKI failure (Maemondo et al., 2010; Mitsudomi et al., 2010; Rosell et al., 2012; Zhou et al., 2011). The response rate of our study is similar to other second line chemotherapy (Stinchcombe & Socinski, 2008). According to the previous phase II study, the response of TS1 plus gemcitabine was 27%, PFS was 4.2 months (95% CI [3.2–5.7]), and OS was 12.9 months (95% CI [10.4–12.7]) (Seto et al., 2010). In our study, the progression free survival was also similar to conventional second line chemotherapy (Fossella, Lynch & Shepherd, 2002). The OS is not significantly different between TTS1 responder and non-responder group, however. This may be due to the fact that OS is affected by many other chemotherapeutic drugs in addition to TTS1. The patients who respond to TTS1 did not mean that they wound respond to other medications. In addition, our patient numbers were not large. The result may show significantly different if there are more patients who could receive TTS1. Since our patients were all treated for at least two different regimens before they received TTS1, the results were impressive. In addition, no grade 3 or higher treatment related toxicities were found in these 28 patients making this regimen a promising treatment option for later line chemotherapy in previously heavily treated NSCLC.

The frequency of *EGFR* mutation is high in Asian patients (Liam, Pang & Poh, 2014; Shi et al., 2015). The patients with NSCLC that harbor *EGFR* mutation is known to respond well to EGFR TKI (Paz-Ares et al., 2017). *EGFR* mutation have been found in gastric cancer also. However, EGFR TKI seems not to respond as well as lung cancer (Dragovich et al., 2006). In our study, the response rate was 23.5% and the disease control rate was 58.8% in patients with *EGFR* mutation. The PFS was 4.9 months. According to previous study of *EGFR* mutated patients, the response rate of 2nd line chemotherapy after receiving first line EGFR-TKI treatment was 24.5% and the PFS was 4.5 months (Tseng et al., 2016). Therefore, our study demonstrated that the response rate and PFS of TTS1 were similar to other regimens for previously treated *EGFR* mutated patients.

On 2014, Liang and his colleagues conducted a meta-analysis and concluded that *EGFR* mutation status also influenced the efficacy of chemotherapy (Liang et al., 2014). One hypothesis is that *EGFR* signaling is associated with cytotoxic chemotherapy induced tumor cell apoptosis (Dixit et al., 1997). Therefore PFS and OS may be affected by the nature of the tumor itself and the effect of other drugs. In our data, the PFS and OS for *EGFR* mutated patients were also higher than those without *EGFR* mutations after receiving TTS1 treatment. This result is compatible with previous studies, too.

There are some limitations in our study. First, it was a retrospective study. Selection bias was possible. Therefore, the data should be interpreted carefully. Second, the number of patients of this study is small. A large scale trial is warranted in the future. Finally, our data are still from patients of Asia. More patients from other races should be enrolled to analyze this regimen.

## Conclusions

In conclusion, TTS1 is an effective regimen for NSCLC previously heavily treated, especially in tumor *EGFR* mutated patients. Paclitaxel plus TS1 is another treatment of choice for NSCLC patients before a more efficient treatment strategy is found.

##  Supplemental Information

10.7717/peerj.7767/supp-1 Supplemental Information 1 Raw data

## Additional Information and Declarations

### Data Availability

The following information was supplied regarding data availability: The raw measurements are available in the Supplemental File.
